# Cattle type and liver abscess occurrence impact aged beef steak instrumental retail color and metabolomics

**DOI:** 10.1093/jas/skag094

**Published:** 2026-04-10

**Authors:** Megan E Eckhardt, Mia B McCracken, Loni W Lucherk, Trent E Schwartz, Emilie C Baker, Ty E Lawrence

**Affiliations:** Department of Agricultural Sciences, West Texas A&M University, Canyon, TX 79016, United States; Department of Agricultural Sciences, West Texas A&M University, Canyon, TX 79016, United States; Department of Agricultural Sciences, West Texas A&M University, Canyon, TX 79016, United States; Department of Agricultural Sciences, West Texas A&M University, Canyon, TX 79016, United States; Department of Agricultural Sciences, West Texas A&M University, Canyon, TX 79016, United States; Department of Agricultural Sciences, West Texas A&M University, Canyon, TX 79016, United States

**Keywords:** discoloration, liver abnormality, metabolism, redness, quality

## Abstract

Beef color may be impacted by many factors, including inherent muscle composition, animal health, antemortem stress, and postmortem processes. The objectives of this study were to determine the associative effects of cattle genetics and liver abscess occurrence upon objective color stability of aged beef *longissimus lumborum* steaks in a simulated retail setting and understand how postmortem muscle metabolism is impacted by cattle genetics and liver health. Strip loins (*n* = 44) were collected from the right side of cattle designated as either native beef or dairy-crosses (NAT or DX; *n* = 22/cattle type). Among cattle phenotypes, half of each set of carcasses were from animals having either a healthy, edible liver or a major liver abscess (*n* = 22/liver outcome; *n* = 11/cattle type-liver outcome combination). The first four anterior steaks were cut and randomly assigned to aging durations of 7, 21, 35, or 49 d, whereas the preceding wedge steak was removed and reserved for metabolomic analysis. Steaks were overwrapped at the completion of aging and evaluated for instrumental color (L*, a*, b*) during a 144-h simulated retail display. Steaks obtained from DX cattle provided a greater range of color measurements, whereas steaks of NAT cattle were intermediate of DX values. Steaks of DX cattle with an edible liver produced the lightest (*P *< 0.01) and least red (*P *< 0.01) colored steaks throughout the display. Steaks of NAT cattle with an edible liver presented the highest (*P *< 0.01) mean redness value at 24 h of display. Steaks from cattle with a liver abscess sustained numerically higher a* values and oxymyoglobin percentages. As would be expected based upon prior literature, the longer the duration of age, the less red (*P *< 0.01) and more discolored (via greatest metmyoglobin percentage; *P *< 0.01) steaks measured. Numerous metabolic pathways (galactose, sucrose, and glutathione metabolism) contributory to glycolysis, indicative of metabolic or oxidative stress, were impacted (*P *< 0.01) by significantly different metabolites among categorical treatment effects, as well as branched-chain amino acid biosynthesis (*P *< 0.01), functional in protein synthesis. This study demonstrated the associative effects of liver abscess outcomes among native and dairy-cross cattle, along with differing postmortem aging times upon beef color and color stability. Likewise, minimizing steak age prior to retail display optimizes the ideal red color preferred by beef consumers.

## Teaser

This research demonstrates that both cattle type and liver health influence beef color and its stability during display, providing new insight into how management and processing factors are expected to affect consumer-perceived meat quality.

## Introduction

Previous literature accredits color as the most critical factor in consumer selection of retail meat products, commonly superseding meat price (Kropf 1980; Hedrick et al 1994; Page et al 2001; Suman et al 2023). Meat discoloration has been well established as a discriminatory attribute by consumers, and as a result, undesired colors have led to extensive economic losses (Ramanathan et al 2022). Muscle color changes from oxymyoglobin to metmyoglobin during retail display are well documented. Intrinsic factors, including breed and health (Wegner et al 2000; Page et al 2001; Brown and Lawrence 2010; Frink 2021), as well as extrinsic factors, including postmortem handling (Mancini and Ramanathan 2014; Colle et al 2015; Eastwood et al 2016), are known to impact biochemical changes of meat products. Variability of muscle composition and metabolic traits specific to fiber types and their unequal proportions amongst breeds of cattle suggests additional implications upon retail color performance (Wegner et al 2000; Page et al 2001; Picard and Gagaoua 2020).

As the population of dairy-cross cattle in the feedlot has risen in response to beef processor market dynamics (Moore 2017), their integration leaves numerous questions regarding industry impact. Most recently, while improved marbling and sustained color stability were documented by Mayer et al (2024) and Frink (2021), respectively, dairy-influenced animals largely create an economic and efficiency deficit in commercial beef processing facilities with regard to their increased liver abscess frequency (Herrick et al 2022; Grimes et al 2024; Taylor et al 2025). Currently minimally documented, Schwartz et al (2025) indicated the sustained preservation of redness among increasing severities of liver abscesses. However, though lacking in literature are the confounding associations of both cattle type as well as liver abscess pathology upon retail color life, nor the biochemical metabolic explanation of these characteristics of aged beef steaks. Thus, the objectives of this study were 1) determine the associative effects of native versus dairy-influenced genetics and liver abscess occurrence upon objective color stability of aged beef *longissimus lumborum* steaks in a simulated retail setting, and 2) understand how postmortem muscle metabolism is impacted by cattle genetics and liver health.

## Materials and methods

Animal Care and Use Committee approval was not obtained for this study because samples were collected post-mortem after animals had been humanely harvested in a USDA-inspected slaughter facility. Foundational data (Schwartz 2023) provided an estimate of expected variability (SEM of 0.82 for 28 h a* value). The target sample size for this experiment was calculated as 12 per treatment or 24 per main effect at α  =  0.01 and β  =  0.90.

### Beef selection, storage, and fabrication

Steers (*n* = 44) were identified during evisceration (0 d) at a commercial beef processing facility in the Texas Panhandle for cattle genetic type (CATTLE; NAT = native; DX = dairy-cross) and liver abscess occurrence (LIVER; EDIBLE = edible liver; ABSCESS = major liver abscess [A+, Elanco Animal Health Liver Check System, Elanco, Greenfield, IN]). Cattle were selected from two feedyards of the same corporate cooperator; no additional background information (use of growth-promoting technologies) was known. Strip loins (IMPS #180; USDA 2020, mean marbling score = 487 ± 112) from the right side of each carcass were obtained and transported to the West Texas A&M University—Caviness Meat Science & Innovation Center. Strip loins were stored and allowed to age in their original vacuum packaging for 6 d at 2–4 °C until steak fabrication.

The four most anterior 1.3 cm-thick steaks (*n* = 176 total steaks) were cut (Treif, Model Lion F, Shelton, CT), individually vacuum-packaged (3 mil thick Clarity Vacuum Pouches, Bunzl Processor Division, Riverside, MO), and stored in the absence of light at 2–4 °C until d 7, 21, 35, or 49 postmortem for retail display. The wedge steak (initial steak slice removed to square the subprimal perpendicular to the long axis prior to steak fabrication) from each strip loin was identified and collected for metabolomic analysis and stored in the same aging conditions as the steaks. Steaks, from anterior to posterior, were ordered and randomly assigned aging treatments (AGE7, AGE21, AGE35, or AGE49) prior to retail display and aged in storing conditions as previously described.

### Experimental treatments and study design

A completely randomized experimental design utilizing a 2 × 2 categorical treatment structure (CATTLE × LIVER) was used to develop four categorical combinations (*n* = 11 strip loins per CATTLE × LIVER combination), with equal representation among each aging treatment (AGE7; AGE21; AGE35; AGE49). Individual animal was considered the experimental unit (*n* = 44; 11 steaks per CATTLE × LIVER for each aging duration), and each steak was evaluated as the sampling unit, with four per animal (*n* = 176).

### Retail display

Upon retail display period at either d 7, 21, 35, or 49 postmortem, vacuum-packaged steaks designated for respective aging treatments (*n* = 44 per retail display period) were unpacked, overwrapped, and displayed in the retail case for 144 h at 2–4 °C. Individual steaks were placed on black expanded polystyrene trays (4S Black Foam Meat Trays, CFK Incorporated, Hantsport, NS, Canada), with absorbent pads (White 4″ × 7″ Absorbent Meat, Fish, and Poultry Pads, Tite-Dri Industries, Boynton Beach, FL), and overwrapped with polyvinyl chloride film (oxygen-permeable polyvinyl chloride fresh meat film; 15,500 to 16,275 cm^3^ O_2_ m^−2^ 24 h^−1^ at 23 °C, E-Z Wrap Crystal Clear Polyvinyl Chloride Wrapping Film, Koch Supplies, Kansas City, MO, USA) using a Winholt Film Wrapper Machine (Model WHSS-1, Winholt Equipment Group, Dallas, TX). Steaks were placed under continuous light-emitting diode lighting (987 ± 415 lux) in a multideck style, open-front case (Multi-Deck Self-Contained Merchandiser, Model OHMA-NRG, Hillphoenix, Conyers, GA) and rotated within the case every 12 h to ensure equal placement opportunity within the multideck case, considering lighting variation between and within levels of the case.

### Retail color evaluation

Simulated retail color evaluation via instrumental measurements and perceptible color (chroma saturation, hue angle, Delta E) calculation parameters were defined in reference to the American Meat Science Association Guidelines for Meat Color Measurements (King et al 2023). A Hunter MiniScan EZ 4500 (Hunter Associates Laboratory, Inc., Reston, Virginia) with a 45˚/0˚ directional viewing geometry, 31.8 mm port, and 25 mm viewed area was used to measure L* (lightness), a* (redness), and b* (yellowness) values, as well as the spectral reflectance values (470, 480, 520, 530, 570, 580, 610, and 700 nm) necessary to calculate percentage oxymyoglobin (OMb), deoxymyoglobin, and metmyoglobin (MMb) using methods defined in Kryzywicki (1979). Every 12 h, measurements were determined at three locations per steak and averaged per time interval during display.

### Metabolomics

The wedge steak was divided into sections, and respective sections were aged individually for 7 or 35 d in the same conditions as retail display-designated steaks and frozen at −28 °C until shipping upon reaching 7 or 35 d of age. Upon shipment to the West Coast Metabolomics Center (University of California, Davis, CA), a 1 g intact muscle sample was obtained from each frozen wedge subsection and placed in 2 mL polypropylene microtubes (Eppendorf Safe-Lock Tubes—Microtube, Fisher Scientific, Waltham, MA) and shipped frozen. Untargeted metabolite analysis was completed using a gas chromatography-mass spectrometry approach according to procedures previously documented by Fiehn (2008) and Kiyimba et al (2023).

### Statistical analysis

Mixed linear models (GLIMMIX) procedures of SAS (version 9.4, SAS Institute, Cary, NC) were utilized to evaluate treatment comparisons, with a pre-set α  =  0.05 (*P *≤ 0.10 accepted as a tendency). Data were analyzed using a completely randomized design with a 2 × 2 × 4 categorical structure. The factorial consisted of the following main effects: two cattle types (NAT or DX), two liver abscess outcomes (EDIBLE or ABSCESS), and four aging durations (AGE7, AGE21, AGE35, or AGE49). Hours of display were also considered a main effect for color analysis and were analyzed as repeated measures using a first-order autoregressive covariance structure. Random effects included the anatomical steak number within each strip loin allocated to the respective aging duration and feedyard origin (the two sampled feedyards consisting of a common corporate cooperator). Marbling score was accounted for as a covariate to control for lean area variance. Metabolomic data were normalized to the mean mTIC across samples and subsequently log_10_-transformed prior to statistical analysis. Significance was determined (α  =  0.05) as previously described among main effects and factorial interactions and further analyzed for pathway impact and principal component analysis (PCA) using MetaboAnalyst (v 6.0). In preparation for using MetaboAnalyst, pathway analysis of treatment interactions prompted modification of the data and treatment format to allow for treatment interactions to cooperate with the MetaboAnalyst one-factor analysis (e.g. CATTLE × LIVER = DXEdible; CATTLE × AGE = NAT35; LIVER × AGE = Abscess7). Results interpreted from MetaboAnalyst were focused upon postmortem bovine muscle evaluation; authors acknowledge the origin of the pathway analyses were originally developed for human medical purposes.

## Results

Among all color and metabolomic characteristics evaluated, no four-way interaction (*P *≥ 0.51) existed among CATTLE × LIVER × AGE × HOUR.

### Instrumental lightness

**Figure 1:**
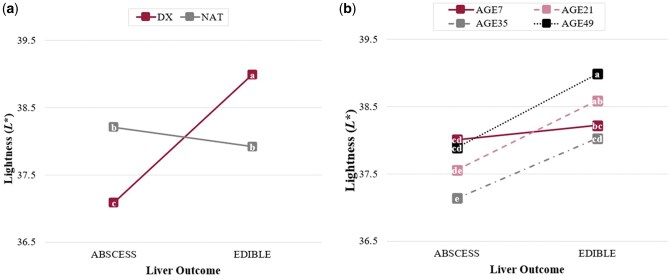
Least square means for objective lightness (L*) values for beef *longissimus lumborum* steaks impacted by a) cattle type^1^ × liver^2^ (*P *< 0.01) and b) age^3^ × liver (*P *= 0.05) interactions. ^1^Cattle types: DX = dairy-cross; NAT = native. ^2^Liver: ABSCESS = major liver abscess (A+, Elanco Animal Health Liver Check System); EDIBLE = healthy liver. ^3^Age: AGE*n* = number of days aged prior to retail display (7, 21, 35, or 49 d).

Instrumental lightness (L*; Figure 1a) values were impacted by the CATTLE × LIVER interaction (*P *< 0.01), in which steaks derived from DX cattle provided the widest range in lightness values. Steaks obtained from DX cattle with an edible liver were the lightest (*P *< 0.01) in color, whereas DX cattle containing a liver abscess presented the darkest (*P *< 0.01) colored steaks. Steaks derived from NAT cattle, regardless of liver abscess outcome (*P *= 0.12), were intermediate of DX steaks. Moreover, the AGE × LIVER interaction (*P *= 0.05) is illustrated in Figure 1b. Except for AGE7 steaks, within each aging duration, steaks derived from cattle with an edible liver presented a lighter (*P *< 0.01) color than steaks from liver-abscessed cattle. No difference (*P *≥ 0.42) in lightness values were observed in steaks aged only 7 d. Beyond AGE7 steaks, no nominal trend was observed among aging duration regardless of liver outcome and lightness values within the interaction, i.e. AGE49 steaks were numerically lighter than AGE21 steaks, which were lighter than AGE35 steaks. Of steaks obtained from cattle with a liver abscess, steaks aged for 7 d tended (*P *= 0.08) to produce greater lightness values than AGE21, similar (*P *= 0.61) to steaks aged 49 d. Similarly, steaks of cattle containing an edible liver and aged 7 d did not differ (*P *≥ 0.15) in lightness from AGE21 and AGE35 steaks. Steaks derived from cattle with an edible liver and aged 49 d produced numerically the lightest values, whereas steaks obtained from cattle with a severe liver abscess and aged 35 d presented numerically the darkest color. While statistical differences in lightness occurred in the current study, it is important to note mean lightness values ranged from 37.09 to 38.99, likely illustrating minimal differences perceivable by the human eye according to Mancini et al (2022), who indicated a 2.10 change in L* value was required for detection of color change in ground beef by trained panelists.

### Instrumental redness

**Figure 2:**
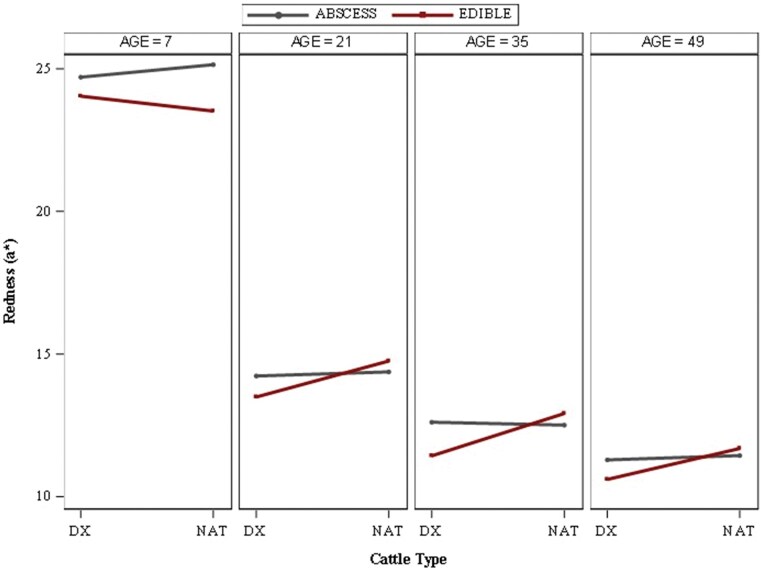
Least square means for objective redness (a*) values for beef *longissimus lumborum* steaks impacted by age^1^ × cattle type^2^ × liver^3^ interaction (*P *< 0.01). ^1^Age: number of days aged prior to retail display (7, 21, 35, or 49 d). ^2^Cattle types: DX = dairy-cross; NAT = native. ^3^Liver: ABSCESS = major liver abscess (A+, Elanco Animal Health Liver Check System); EDIBLE = healthy liver.

**Figure 3:**
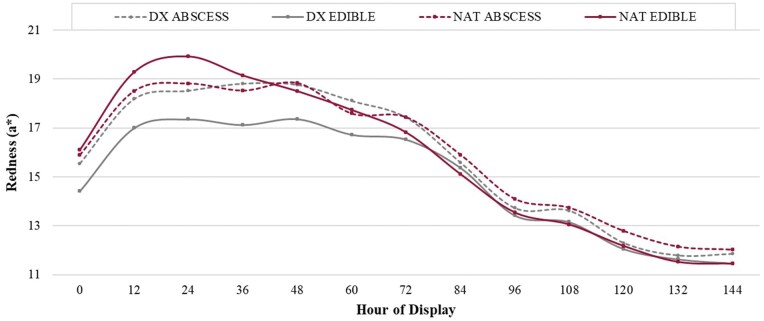
Least square means for objective redness (a*) values for beef *longissimus lumborum* steaks impacted by the cattle type^1^ × liver^2^ interaction across hours of display (*P *< 0.01). ^1^Cattle types: DX = dairy-cross; NAT = native ^2^Liver: ABSCESS = major liver abscess (A+, Elanco Animal Health Liver Check System); EDIBLE = healthy liver.

Two three-way interactions (*P *< 0.01; Figure 2, AGE × CATTLE × LIVER; Figure 3, HOUR × CATTLE × LIVER) were observed for redness (a*) values. Steaks aged for only 7 d were notably the reddest (*P *< 0.01) among all steaks observed within the study. Steaks aged for 7, 21, and 35 d all differed (*P *≤ 0.03) in redness values from one another, where redness continued to decline the longer steaks were aged (a* values: AGE7 > AGE21 > AGE35). Steaks aged for 7 or 21 d were redder (*P *< 0.01) in color than AGE49 steaks. All AGE35 steaks produced redder (*P *< 0.01) values than AGE49 steaks, except for AGE35 steaks obtained from DX cattle with an edible liver, which were similar (*P *≥ 0.34) to most AGE49 steaks. Within each aging duration, steaks aged for 21, 35, and 49 d, each cattle-liver abscess categorical combination produced similar numerical trends among redness values. For instance, steaks derived from NAT cattle with an edible liver presented the highest numerical a* values, followed by steaks of cattle with a liver abscess, and finally, steaks from DX cattle with an edible liver recorded the lowest (*P *< 0.01) redness values among aging duration. Steaks from NAT or abscessed-liver cattle did not differ in redness when aged 21–49 d. The primary difference in redness performance of steaks aged 7 d pertains to steaks derived from NAT cattle with an edible liver, of which tended to produce a lower (*P *= 0.06) redness value than steaks of DX cattle with an edible liver and lower (*P *< 0.01) than all other treatment combinations, which is opposite of the other three aging durations. Moreover, AGE7 steaks obtained from cattle with a liver abscess were redder (*P *< 0.01) than steaks of edible liver cattle.

From 0–48 h of display, steaks derived from DX cattle with an edible liver produced the least red (*P *≤ 0.01) color. Initially (0 h), all other steaks were similar (*P *≥ 0.23) in redness until steaks obtained from NAT cattle with an edible liver produced the greatest peak in redness (*P *≤ 0.01) at 24 h of display, at which point steaks derived from cattle with a liver abscess did not differ (*P *= 0.53) from one another, but produced intermediate values compared to steaks from edible liver-cattle. From 96 h and beyond, all treatment combinations remained similar (*P *≥ 0.16) to one another in redness but steadily declined over time. Prior literature discloses an a* value of approximately 14.50 (Holman et al 2017) as the minimum threshold for consumer acceptability of redness; all steaks evaluated for the hour-three-way interaction fell below this threshold (unacceptable) after 84 h of simulated retail display. Notably at 0 h, only steaks obtained from DX cattle with an edible liver produced redness values below this threshold before increasing over the next 12 h.

### Instrumental yellowness

Instrumental b* values differed among the CATTLE × LIVER × AGE interaction (*P *< 0.01; not presented in tabular form), in which steaks aged for 7 d reported higher b* (more yellow; *P *< 0.01) than AGE21 steaks, which were also greater (*P *< 0.01) than AGE35 and AGE49 steaks. Specifically, AGE7 steaks derived from NAT cattle with a liver abscess presented the greatest (*P *≤ 0.05) b* values, and those from NAT cattle with an edible liver tended (*P *≥ 0.06) to produce the lowest b* values.

No distinguishable trend among b* could be identified among the CATTLE × LIVER × HOUR interaction (*P *< 0.01; not illustrated or included in tabular form), though all steaks began and finished the simulated retail display with similar b* values (*P *≥ 0.15). Notably, steaks of NAT cattle with an edible liver peaked with the greatest (*P *< 0.01) b* value at 24 h compared to all other cattle types and liver abscess presence combinations.

### Calculated myoglobin percentages

**Figure 4:**
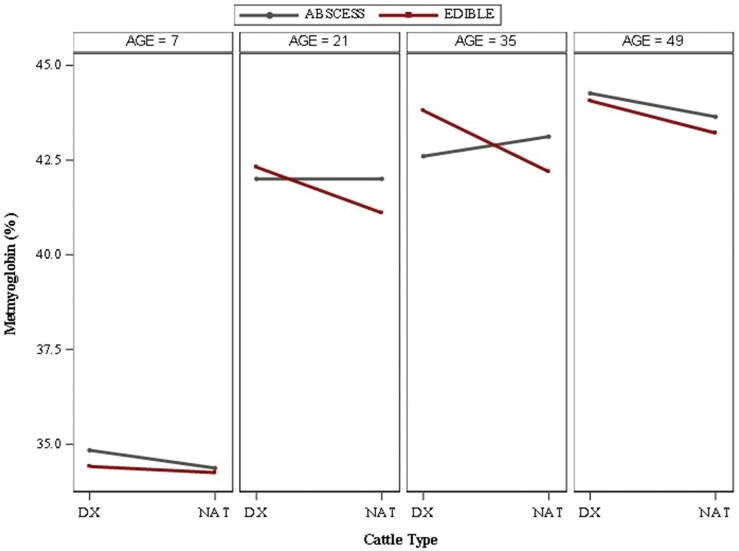
Least square means for percentage metmyoglobin for beef *longissimus lumborum* steaks impacted by age^1^ × cattle type^2^ × liver^3^ interaction (*P *< 0.01). ^1^Age: number of days aged prior to retail display (7, 21, 35, or 49 d). ^2^Cattle types: DX = dairy-cross; NAT = native. ^3^Liver: ABSCESS = major liver abscess (A+, Elanco Animal Health Liver Check System); EDIBLE = healthy liver.

As of function of redness values previously described, steaks aged only 7 d prior to display produced the lowest mean percentage of MMb (<35%; *P *< 0.01), as demonstrated in Figure 4, depicting the AGE × CATTLE × LIVER interaction (*P *< 0.01). Cattle type and liver abscess presence did not impact AGE7 MMb percentages (*P *≥ 0.12). All other aging durations produced mean MMb percentages greater than 40% and generally increased as steak age increased prior to display. Steaks of AGE21 and AGE35 performed similarly among MMb trends for cattle type and liver abscess occurrence, in which steaks from DX cattle with an edible liver produced a numerically greater percentage of MMb than steaks from NAT cattle with an edible liver, whereas steaks from cattle with a major liver abscess remained intermediate to edible liver-steak values. Among AGE21 steaks, only steaks derived from NAT cattle with an edible liver differed from all other AGE21 steaks, producing a lower (*P *≤ 0.02) mean MMb percentage. Within AGE49 steaks, those derived from DX cattle produced higher (*P *≤ 0.03) mean MMb than steaks of NAT cattle with an edible liver, whereas steaks from NAT cattle with a liver abscess remained intermediate (*P *≥ 0.12).

As previously well-established among literature (Mancini and Ramanathan 2014; Frink 2021; Foraker et al 2022; Ramanathan et al 2022), hour impacted percentage MMb (*P *< 0.01; data not illustrated or included in tabular form), initially decreasing upon placement in retail display (0–12 h, *P *< 0.01) before increasing throughout the duration of display (beginning at h 60 until h 108, *P *< 0.01) and plateauing at 108 h through 114 h (*P *≥ 0.10).

**Figure 5:**
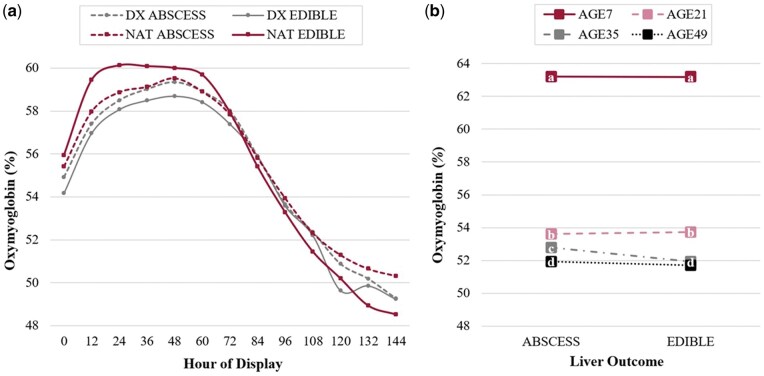
Least square means for percentage oxymyoglobin for beef *longissimus lumborum* steaks impacted by a) cattle type^1^ × liver^2^ across hours of display (*P *= 0.04) and b) age^3^ × liver (*P *= 0.03) interactions. ^1^Cattle types: DX = dairy-cross; NAT = native. ^2^Liver: ABSCESS = major liver abscess (A+, Elanco Animal Health Liver Check System); EDIBLE = healthy liver. ^3^Age: AGE*n* = number of days aged prior to retail display (7, 21, 35, or 49 d).

As would be expected, OMb percentages performed similarly to instrumental redness results regarding the CATTLE × LIVER interaction across the duration of retail display (HOUR; *P *= 0.04). Illustrated in Figure 5a, OMb percentages for all steaks at h 0 did not differ (*P *≥ 0.13) until h 12–24, in which steaks derived from NAT cattle with an edible liver produced the highest (*P *≤ 0.05) percentage of OMb, indicative of a more cherry-red colored steak. In general, steaks increased in OMb percentage from h 0 to 36 (*P *< 0.01), before plateauing (h 36–72; *P *≥ 0.09) and then declining, beginning at h 72 (*P *≤ 0.02).

Reflective of MMb, impacted by aging duration as well as the AGE × LIVER interaction (*P *= 0.03) included in Figure 5b, illustrates the remarkably higher (*P *< 0.01) percentage of OMb in AGE7 steaks compared to all other steaks (no difference among LIVER outcome; *P *= 0.95). A reverse trend discussed for MMb, OMb percentages declined (*P *< 0.01) as duration of age prior to retail display increased, except no difference in OMb (*P *= 0.36) was detected among steaks from cattle with an edible liver aged for either 35 or 49 d. No difference (*P *≥ 0.32) in percentage OMb was observed within each AGE-duration, except steaks aged 35 d, in which steaks derived from cattle with a major liver abscess produced a higher (*P *< 0.01) percentage OMb than their healthy counterparts.

### Chroma saturation, hue angle, and Delta E

Chroma and hue derived from instrumental color measurements each produced an AGE × CATTLE × LIVER interaction (*P *< 0.01; data not presented in tabular form). Steaks aged for 7 d prior to retail display produced greater (*P *< 0.01) chroma saturation and smaller hue angle values, indicating a more saturated, intense red color when compared to other aging durations. Decreasing chroma saturation and increasing hue numerically trended accordingly as aging duration increased, thus less saturated or intense in red color and likely indicative of greater MMb formation (King et al 2023). Specifically, AGE21 steaks presented more saturated color (greater chroma values; *P *≤ 0.01), whereas chroma values continued to decline numerically from AGE35 to AGE49 steaks, though overlapping statistically. For steaks aged at least 21 d, within each age duration, steaks obtained from cattle with a liver abscess presented no difference (*P *≥ 0.26) in saturation among cattle types and were intermediate to chroma values of steaks of healthy-liver cattle. Steaks obtained from NAT cattle with a major liver abscess and aged ≥ 21 d revealed greater (*P *< 0.01) chroma values than their DX counterparts.

Similarly, and complementary to chroma data, no difference (*P *≥ 0.24) was determined for hue angle values of steaks obtained from cattle with a major liver abscess. Likewise, hue values for steaks (AGE21–49) of liver-abscessed cattle bisected hue values of cattle with an edible liver, in which DX cattle with an edible liver presented a greater (*P *< 0.01) mean hue angle, indicating less redness or greater MMb formation when compared to NAT cattle with an edible liver.

Mean color change (ΔE) for both the CATTLE × LIVER interaction as well as aging duration are included in Table 1. Across the entire duration of display (144 h), no difference (*P *≥ 0.32) was reported among the CATTLE × LIVER interaction nor aging durations. Upon analysis of segmenting display periods: 0–48 h, 48–96 h, and 96–144 h, the documented ΔE trend numerically increased among segments of display from the beginning to the end of display. Generally, color change of steaks by CATTLE × LIVER did not differ (*P *≥ 0.30; 0–144 h, 0–48 h, and 96–144 h of display), except that color change differences (*P *= 0.05) were detected among CATTLE × LIVER steaks during the 48–96 h duration. Native cattle produced steaks with the greatest (EDIBLE) and least (ABSCESS) color change, indicating steaks of NAT cattle with a major liver abscess may promote better color stability (*P *= 0.04) under a sustained duration of display. Steaks from DX cattle were intermediate (*P *≥ 0.13) of the respective NAT steak ΔE values. Color change (ΔE) did not differ (*P *≥ 0.30) among cattle type and liver abscess occurrence through the duration of 0–48 h and 96–144 h of display. Among aging durations, steaks aged for 21 d experienced the greatest (*P *≤ 0.01) color change from 0–48 h, no difference occurred from 48–96 h (*P *= 0.11), and at 96–144 h, notably AGE7 steaks experienced the greatest (*P *≤ 0.01) change in color.

**Table 1:** Change in color (ΔE) of beef *longissimus lumborum* steaks over the 144-h duration of display, as well as segmented durations (0–48, 48–96, 96–144 h).

	Delta E^1^			
Cattle × liver^2^	0–144 h	0–48 h	48–96 h	96–144 h
**Native**				
**Abscess**	7.49	3.02	4.38^b^	7.30
**Edible**	7.49	2.64	5.96^a^	8.14
**Dairy-cross**				
**Abscess**	8.56	3.06	5.23^ab^	7.36
**Edible**	8.34	3.29	4.75^ab^	7.55
**SEM^3^**	1.14	0.32	0.59	0.98
***P*-value**	0.87	0.30	0.05	0.68
**Age**				
**7 d**	7.67	2.34^b^	4.28	10.43^a^
**21 d**	8.99	3.97^a^	6.03	7.47^b^
**35 d**	8.02	2.89^b^	5.25	6.32^b^
**49 d**	7.20	2.82^b^	4.77	6.14^b^
**SEM^3^**	0.98	0.29	0.54	0.91
***P*-value**	0.32	0.01	0.11	< 0.01

1 Delta E (ΔE) = [(ΔL*)^2^ + (Δa*)^2^ + (Δb*)^2^]^0.5^.

2 Liver: Abscess = major liver abscess (A+, Elanco Animal Health Liver Check System); Edible = healthy liver.

3 SEM = largest standard error of the mean (SEM) reported for least squares means.

### Metabolomics

Untargeted metabolomic analysis was accomplished via gas chromatography-mass spectrometry. A total of 160 known compounds were identified for steaks assessed for significant (*P *≤ 0.05) distinctions among aging (AGE7 versus AGE35), cattle type, and/or liver abscess occurrence. Of total known compounds, numerous metabolites were different (*P *≤ 0.05; included in Supplemental Table 1) in the overall analysis for respective main effects and interaction differences: AGE (53), CATTLE (6), LIVER (14), CATTLE × LIVER (33), AGE × CATTLE (59), AGE × LIVER (12), and the three-way interaction of AGE × CATTLE × LIVER (5). Many metabolic pathways, listed in Table 2, were associated with glycolysis or other carbohydrate metabolism, as well as a few involved with amino acid biosynthesis and peptide metabolism. Too few associated metabolites were identified among the cattle type main effect to perform any meaningful pathway analysis; therefore, none are included.

**Table 2:** Significantly impacted metabolic functions or pathways, impacted by significantly different metabolites among aged beef *longissimus lumborum* steaks^1^ of differing duration aged, liver abscess presence, and/or cattle type (*n* = 88 steaks; *n* = 44/main effect).

Function/pathway and metabolites	Holm adjusted *P*-value	FDR^2^
**Age**		
**Galactose metabolism**	<0.01	<0.01
**Metabolites: glycerol, sucrose, fructose, mannose, glucose-1-phosphate, lactose**		
**Glycogen & sucrose metabolism**	0.01	0.01
**Metabolites: maltose, glucose-1-phosphate, sucrose, fructose**		
**Liver**		
**Valine, leucine, and isoleucine biosynthesis**	<0.01	<0.01
**Metabolites: threonine, isoleucine, valine**		
**Cattle × liver**		
**Glycogen and sucrose metabolism**	<0.01	<0.01
**Metabolites: maltose, cellobiose, glucose-6-phosphate, fructose, glucose**		
**Cattle × age**		
**Valine, leucine, and isoleucine biosynthesis**	<0.01	<0.01
**Metabolites: threonine, isoleucine, leucine, valine**		
**Liver × age**		
**Glutathione metabolism**	<0.01	<0.01
**Metabolites: ornithine, putrescine, 5-oxoproline**		

1 Main effect and interaction classifications: age = 7 d or 35 d age duration; cattle type = dairy-cross or native; liver = major liver abscess (A+, Elanco Animal Health Liver Check System) or edible liver (healthy)

2 False discovery rate (FDR) set at <0.05, with Fisher’s LSD set at *P *< 0.05 to determine significance.

**Figure 6:**
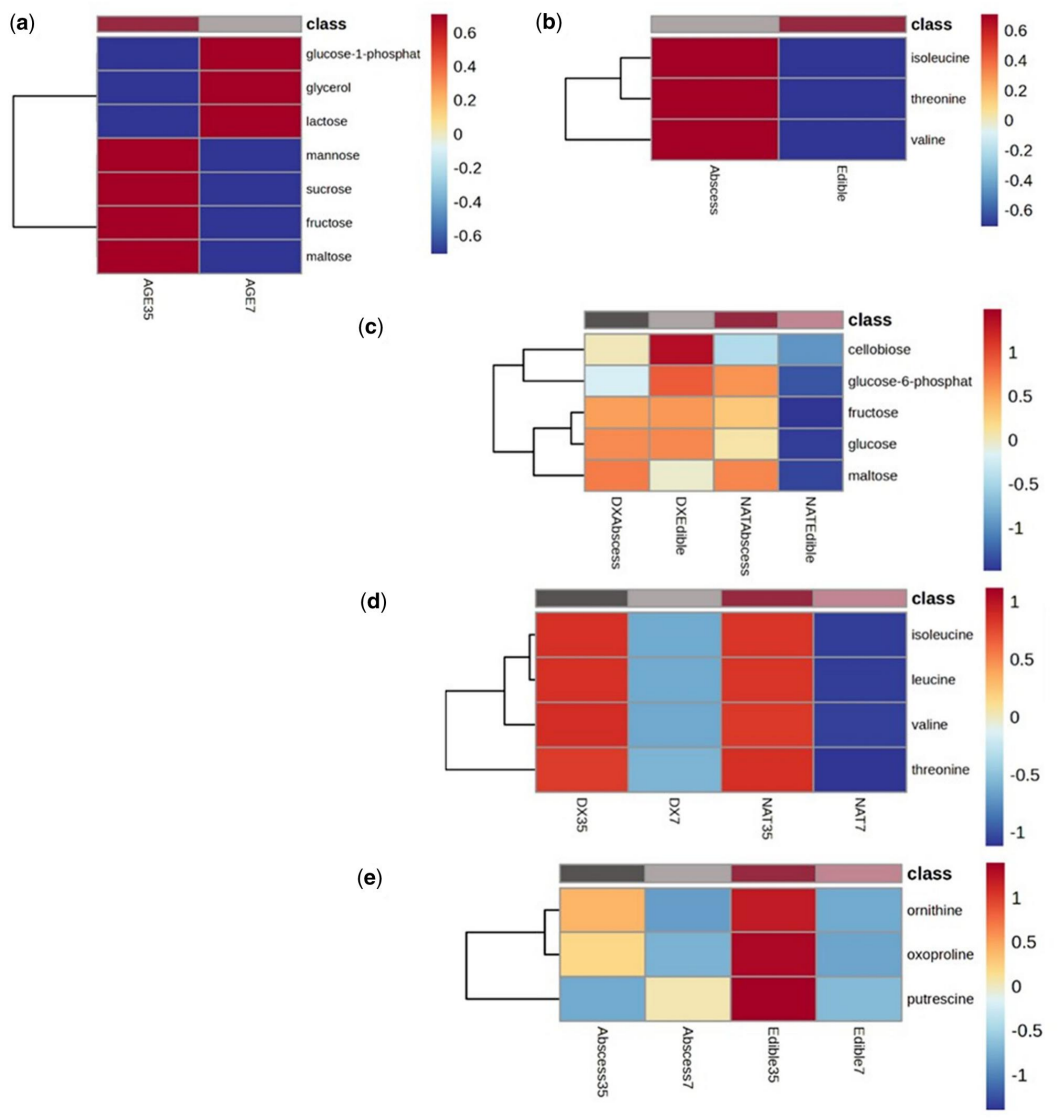
Heat map demonstrating the normalized intensity values of metabolites significantly impacting metabolic functions or pathways in beef *longissimus lumborum* steaks^1^ among aging duration, liver abscess presence, and/or cattle type. Higher, intermediate, and lower abundance is indicated by the 1, 0, -1 scale provided, respectively. a) Age, b) liver, c) cattle type × liver, d) cattle type × age, and e) liver × age. ^1^Main effect and interaction classifications: AGE7 = 7 d age; AGE35 = 35 d age; liver or cattle type followed by 7 or 35 indicate duration of age; DX = dairy-cross; NAT = native; Abscess = major liver abscess (A+, Elanco Animal Health Liver Check System); Edible = healthy liver.

Both galactose metabolism, as well as glycogen and sucrose metabolism were impacted (*P *≤ 0.01) by aging duration. A heat map demonstrating only the normalized intensities of significantly different metabolites that impacted metabolic functions or pathways are illustrated in Figure 6. Galactose metabolism was impacted as a result of increased abundance of mannose, sucrose, fructose, and maltose, concomitant with lower abundance of glucose-1-phosphate, glycerol, and lactose. All three significant metabolites impacting amino acids synthesis (*P *< 0.01) among steaks obtained from cattle with a major liver abscess were detected at a higher abundance when compared to steaks derived for cattle with a healthy liver.

Among the CATTLE × LIVER interaction, glycogen and sucrose metabolism were significantly impacted (*P *< 0.01) functions as a result of significantly different metabolites (maltose, cellobiose, glucose-6-phosphate, fructose, and glucose) among cattle types and liver abscess occurrence outcomes. Remarkably, all metabolites were detected in the lowest abundance for steaks derived from NAT cattle with an edible liver, relative to all other CATTLE × LIVER categorical combinations. This is particularly interesting as steaks derived from such cattle sustained the highest mean percentage OMb, as previously discussed in Figure 5. Furthermore, in accordance with lower OMb percentages, steaks from all DX cattle and NAT cattle with a liver abscess, the relative abundance of the respective metabolites tended to exist higher. Thus, suggesting increased abundance of the respective metabolites likely decreases OMb stability, depreciating retail case-life and possibly consumer acceptability.

In brief, the CATTLE × AGE interaction impacted (*P *< 0.01) the biosynthesis of valine, leucine, and isoleucine via threonine, isoleucine, leucine, and valine. Predominantly driven by aging duration, steaks aged for 35 d experienced an increased abundance of all significantly different metabolites, whereas AGE7 steaks were lower in abundance, especially AGE7 steaks derived from NAT cattle.

Significantly different metabolites (ornithine, putrescine, 5-oxoproline) impacting glutathione metabolism (*P *< 0.01) among the liver abscess occurrence and aging duration combination illicit similar tendencies as witnessed in OMb percentage results (Figure 5). Steaks aged for 7 d (regardless of liver outcome) as well as AGE35 steaks obtained from cattle with a major liver abscess produced an intermediate to lower abundance of the respective metabolites (of glutathione metabolism) compared to the higher abundance reported for steaks obtained from cattle with an edible liver and aged for 35 d. Such steaks also exhibited greater OMb percentages (more red in color, similar general trend in instrumental redness) when compared to AGE35 steaks obtained from cattle with an edible liver. Results indicated an increased abundance of glutathione metabolism compounds may be associated with increased rates of oxidation, reducing OMb percentages, as witnessed in AGE35 steaks from cattle with an edible liver.

**Figure 7:**
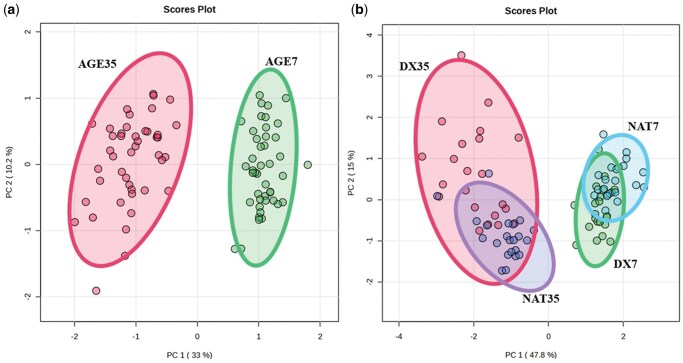
Principal component analysis of significantly different metabolites of beef *longissimus lumborum* steaks among a) aging^1^ duration and b) cattle type × age interaction.^2^ ^1^Age: AGE*n* = number of days aged prior to retail display (7 or 35 d). ^2^Cattle type *×* age interaction: DX = dairy-cross; NAT = native; followed by aging duration (7 or 35 d). Illustrations not included for the following main effects and interactions: cattle type (*R*^2^ = 0.01, *P* = 0.72); liver (*R*^2^ = 0.04, *P* = 0.14); cattle type × liver (*R*^2^ = 0.19, *P* = 0.01); liver × age (*R*^2^ = 0.46, *P* < 0.01); cattle type × liver × age (*R*^2^ = 0.68, *P* < 0.01).

Demonstrated in Figure 7 are the principal component analyses (PCA) including the distinct separation of significantly different metabolites among the aging duration main effect (R^2^ = 0.71, *P *< 0.01) and the interaction between cattle type and aging duration (R^2^ = 0.80, *P *< 0.01). While distinct separation was highly driven by aging duration, among cattle type, an inverse relationship existed for NAT and DX cattle for AGE7 and AGE 35 steaks, illustrating the interaction within the PCA plot. All other main effects or factorial interactions were not included as such groups lacked sufficient separation (R^2^ ≤ 0.68), beyond the separation predominantly driven by aging duration.

## Discussion

Instrumental redness and lightness among aged beef are well documented across numerous durations of aging. As aging duration increases, a subsequent reduction in mitochondrial activity and metmyoglobin-reducing activity occurs, thus, the faster the deterioration of redness and lightness, even discerning redness or lightness upon initial bloom when placed in the retail case (Mancini and Ramanathan 2014; Colle et al 2015). The current study included aging durations ranging from 7 to 49 d prior to simulated retail display to encompass the majority of the industry spectrum (Martinez et al 2017). Recorded in 2015, the age of steaks prior to display at retail establishments ranged from 6 to 102 d for retail (mean age 25.9 d) and 3 to 91 d for foodservice establishments (mean age 31.5 d; Martinez et al 2017). Similar to the 0 d aged steaks of the Mancini and Ramanathan (2014) study, the 7 d aged steaks in the present study indicate a small or limited aging duration promotes greater redness and higher percentages of oxymyoglobin sustained during retail display, likely as a result of higher metmyoglobin-reducing activity and greater mitochondrial activity compared to longer aging durations. Initial redness of steaks upon simulated retail display as aging duration increased not only likely decreased in redness as a result of extended aging (7 to 49 d) but also as a result of physical product form while aging. Steaks evaluated in the current study were aged in steak form (versus subprimal form), individually in vacuum packages, which may have promoted additional redness deterioration throughout display, compared to what steaks cut from a subprimal post-age may have displayed. Eastwood et al (2016) reported no difference in lightness among aged steaks versus steaks cut from aged subprimals but reported redder values for display product derived from an aged subprimal, rather than the individually aged steak.

Cattle genetics have previously been reported to impact beef color, primarily driven by muscle fiber composition and inherent muscle metabolism (Wegner et al 2000; Page et al 2001; Picard and Gagaoua 2020; Frink 2021). The degree of impact is relative to respective breed comparisons. For instance, Wegner et al (2000) disclosed differing proportions of muscle fiber composition, brightness, and redness of the m. *semitendinosus* in German Angus, Galloway, Holstein Friesian, and Belgian Blue cattle—in which Belgian Blue cattle tended to differ the greatest from all other breed comparisons regarding fiber type frequencies and overall lightness. Likewise, Page et al (2001) reported dairy-type cattle to produce a darker, less-red muscle color when compared to native and Brahman beef. Picard and Gagaoua (2020) would reasonably suggest that while differences among distinctly different breed types promote differences in muscle fiber composition, the crossing of such breeds would yield an intermediate—which brings about beef × dairy-crosses and their resulting muscle fiber composition and color characteristics. Previous research suggests dairy breeds contain a greater proportion of red (Type-I) muscle fibers, more oxidative in nature, thus more red in color (Picard and Gagaoua 2020). Some have accepted that dairy-influenced animals promote greater color perception or sustainability, though literature generally suggests otherwise—where no difference exists among traditional beef breeds and dairy-influenced beef (Foraker et al 2022). Frink (2021) reported a remarkable difference in lightness and redness of strip loin steaks among full-blood dairy-type cattle versus native beef, though dairy-influenced animals (beef × dairy-cross), while intermediate, more closely resembled the native beef color. While the respective dairy-cross steaks tended to be more red in color compared to the native beef, this slightly differs from the present study. Specifically, considering how the presence of a liver abscess influenced the color of steaks derived from DX cattle containing a liver abscess promoted a darker-red color, whereas DX cattle with an edible liver were associated with a lighter-red-colored steak. Foraker et al (2022), on the other hand, reported no color differences of strip loin steaks among varying degrees of dairy influence when compared to a full beef-type animal.

Discernment among cattle type influence may be explained by unknown bias toward cattle selection with either healthy or abscessed livers. For instance, steaks of the current study obtained from dairy-influenced cattle with a major liver abscess produced adequate redness (a*) values, but when coupled with the darkest (L*) values, this promoted a much darker, more intense red color. This becomes concerning considering the opportunity for misclassifying dairy-influenced animals as dark cutting, likely resulting in a downgraded and discounted carcass (Bass et al 2008; USDA 2017). Dairy-influenced animals lacking liver abscesses (healthy, edible liver) represented the opposite redness and lightness association. This reveals another incentive beyond those mentioned by previous literature to reduce liver abscesses in beef cattle, particularly in dairy-influenced animals (Brown and Lawrence 2010; Herrick et al 2022; Taylor et al 2025). Likewise, Schwartz et al (2025) mention steaks from dairy crosses with a major liver abscess promoted a redder, more saturated (chroma) steak color over the course of a 5 d display when compared to steaks from dairy crosses with normal, healthy livers. While existing research discusses the association of liver abscess presence and severity upon impaired carcass merit (quality grade, yield grade, gross carcass value, and other associated losses; Brown and Lawrence 2010; Grimes et al 2024; Taylor et al 2025), as well as other beef quality characteristics (sensory characteristics; McCoy et al 2017), literature lacks discussion associating liver abscess occurrence to beef color performance.

Though much improved over time, through the use of numerous pharmaceutical or nutritional remedies, liver abscess prevalence remains high in dairy-influenced animals comparative to native beef breeds (Grimes et al 2024; Taylor et al 2025). The liver serves as a metabolic “hub” for an animal, metabolically connecting tissues across the body—including skeletal and adipose tissues (Rui 2014). Therefore, presence of a major liver abscess impairs liver function, promoting metabolic stress (Rui 2014), likely cascading to metabolic and oxidative stress in skeletal muscle altering beef color.

Ramanathan et al (2023) explain the critical role in biochemical changes driven by mitochondrial and metabolic functions to better understand the change in shelf-life and impact upon other eating characteristics of meat—namely, metabolites in which the over- or under-abundance impacts the tricarboxylic acid (TCA) cycle and glycolytic pathways. In dark-cutting beef, with a stark difference in meat color appearance, primarily driven by antemortem stress and a depleted glycolytic reservoir, a lower abundance of glycolytic metabolites, including glucose-6-phosphate and glucose-1-phosphate, among others, was reported (Ramanathan et al 2020). As previously documented, both aged beef and dark-cutting beef lack the optimal formation of bright cherry-red beef color and do not sustain color through duration or retail display (Wu et al 2020). Similarly, in the current study, steaks aged for 35 d versus 7 d contained a lower abundance of glucose-1-phosphate among other significantly different metabolites impacting both galactose metabolism as well as glycogen and sucrose metabolism, coinciding with a reduction in redness and color stability during the simulated display. However, steaks from NAT cattle with an edible liver also exhibited a lower abundance of metabolites (including glucose-6-phosphate) noted to affect glucose metabolism yet produced the lightest and reddest steak at the beginning of the display, contrary to expectations corresponding to reduced red or increased metmyoglobin.  (Huntington 1990)  discusses differences in metabolic performance between dairy and NAT beef-type cattle, thus impacting the metabolic capacity of glycogen and other carbohydrates. Existing data implies dairy-influenced cattle contain a greater proportion of glucose produced by the liver and thus a greater glycogen content in muscle. Contrary, liver tissue failure, including failure brought upon by abscessation, impedes glycogenesis of the liver and thus reduces stores of muscle glycogen (Chakravarthy et al 2020). Increased levels of muscle glycogen allow for a brighter, more optimal color development through postmortem pH decline, while decreased muscle glycogen stores hinder the development of bright, cherry-red color formation and lead toward darker, more intense red color formation (Miller et al 1987).

Branched chain amino acids (BCAA; valine, leucine, and isoleucine) are essential amino acids and must be supplied via dietary means and eventually reside in skeletal muscle after transportation by the bloodstream (Zhang et al 2017). Such amino acids have been associated with glucose metabolism and intestinal barrier function among other systemic functions (Zhang et al 2017). The BCAA metabolites are later catabolized by a series of enzymatic reactions to participate in the TCA cycle. In both circumstances in which the BCAA biosynthesis was deemed significantly impacted by metabolites in the current study, the contributing amino acids were in higher abundance for steaks from liver abscessed cattle compared to healthy liver cattle, as well as for steaks aged for 35 d among the CATTLE × AGE interactions. As threonine abundance was reported greater, this in return indirectly disrupts BCAA catabolism and the TCA cycle via nitrogen imbalance and nutrient signaling (Tang et al 2021).

Glutathione is predominantly synthesized in the liver and is commonly referred to as the “master antioxidant,” and provides a considerable role in protecting against oxidative stress (Bilinsky et al 2015). Lower abundance of ornithine, putrescine, and 5-oxoproline likely corresponds to reduced overall oxidative stress signaling and depressed glutathione synthesis due to a series of cascading disfunctions among the pathways, primarily driven by 5-oxoproline (Palekar et al 1974; Pegg 2006). Recognizing a lower abundance of the previously mentioned metabolites, especially in steaks aged only 7 d (regardless of liver outcome), as well as steaks of liver-abscessed cattle aged for 35 d, the respective steaks are indicative of reduced overall oxidative stress signaling, which may also coincide with the production of greater percentages of OMb (refer to Figure 5b) when compared to steaks obtained from cattle containing an edible liver, aged 35 d.

Postmortem pH decline is another critical biochemical factor influencing beef color and may have contributed to the variation observed in the present study; however, pH was not measured as part of our data collection, and therefore its role can only be inferred indirectly through color traits and metabolic profiles. The extent and rate of pH decline are largely governed by muscle glycogen availability and downstream glycolytic flux, which regulate mitochondrial oxygen consumption and the formation of oxymyoglobin on the lean surface. When glycogen reserves are limited, muscles typically exhibit a higher ultimate pH, reduced bloom development, and a darker, more intense red surface due to restricted oxymyoglobin formation and increased presence of deoxygenated myoglobin, as is commonly observed in dark‑cutting beef. This framework provides a biologically plausible explanation for the darker, more intense red appearance of steaks from dairy‑cross cattle with major liver abscesses, as impaired liver function could restrict glycogen synthesis and moderate postmortem pH decline. Conversely, the lighter and brighter steaks from cattle with healthy livers align with conditions that would normally support more complete glycolysis and lower ultimate pH. Likewise, the reduced abundance of glycolytic intermediates observed in longer‑aged steaks (e.g. lower glucose‑1‑phosphate) is consistent with reduced biochemical capacity to support favorable pH‑related color development, particularly during bloom and early display.

In summary, this study demonstrated the confounding association of cattle type and liver abscess occurrence upon beef color and color stability, specifically the variance in lightness and redness of steaks obtained from dairy-cross animals of differing liver health. Likewise, minimizing steak age prior to displaying elicits optimal redness of beef steaks, also narrowing differences in color among cattle type and liver health. Additional days of aging promote further degradation of beef retail color, likely exaggerating the inherent color characteristics promoted by cattle type and liver health. Improving liver health of feedlot cattle may help improve the consistency and uniformity of retail steaks.

## Supplementary Material

skag094_Supplementary_Data

## Abbreviations

ΔE: change in instrumental color
*a**: instrumental unit for redness
*b**: instrumental unit for yellowness
*L**: instrumental unit for lightness
MMb: metmyoglobin
OMb: oxymyoglobin
PCA: principle component analysis
