# Supplementary material for: Cattle type and liver abscess occurrence impact aged beef steak instrumental retail color and metabolomics
Source: J Anim Sci. 2026 Apr 10;104:skag094. doi: 10.1093/jas/skag094 (PMC13091092; doi:10.1093/jas/skag094)
Supplement: skag094_Supplementary_Data [file skag094_supplementary_data.docx]

| **Supplemental Table 1.** Metabolites that differed among aged beef *longissimus lumborum* steaks^1^ of differing duration aged, liver abscess presence, or cattle type (n = 88 steaks; n = 44/main effect). | | | |
| --- | --- | --- | --- |
| **Age** | **Age (continued)** | **Age × Cattle (continued)** | **Age × Liver (continued)** |
| Xylulose** | 2-deoxypentitol* | N-carbamoylaspartate* | Lactamide* |
| Xylose** | 2,3-dihydroxypyridine** | N-acetylneuraminic acid* | Lycyl-proline* |
| Xanthine** | 1-monopalmitin** | N-acetylglucosamine-6-phosphate* | Glycyl-glycine* |
| Trans-4-hydroxyproline* | 1-hexadecanol** |  | Beta-alanine* |
| Tagatose** |  | Methionine sulfoxide** | 2-hydroxyvaleric acid* |
| Sucrose** | **Cattle** | Methionine** |  |
| Serotonin** | Serotonin** | Mannitol** | **Cattle × Liver** |
| Salicylic acid* | Pinitol** | Lysine** | Tocopherol gamma** |
| Ribose** | N-acetylmannosamine* | Leucine** | Tocopherol alpha** |
| Ribonic acid** | 2-monoolein* | Lactamide* | Tagatose** |
| Pinitol** | 2-hydroxyisocaproic acid* | Isothreonic acid* | Sophorose** |
| Phenylethylamine** | 1-kestose* | Isoleucine** | Shikimic acid* |
| Pentadecanoic acid** |  | Inositol-4-monophosphate** | Salicylic acid* |
| Parabanic acid** | **Liver** | Inosine-5-monophosphate** | Ribulose-5-phosphate* |
| Pantothenic acid* | Valine* | Inosine** | Ribose-5-phosphate** |
| Octadecanol** | Trans-4-hydroxyproline* | Indole-3-lactate** | Ribose* |
| Nicotinic acid** | Threonine* | Hypoxanthine** | Proline* |
| Montanic acid* | Sucrose* | Guanosine* | Phthalic acid* |
| Mannose-6-phosphate** | Serine* | Glycyl-proline** | Mannose** |
| Mannose** | Phenylethylamine* | Glycyl-tyrosine* | Maltose* |
| Maltose* | N-methyl-UMP* | Glycine* | Malic acid** |
| Maleic acid** | Mannitol** | Glucose-6-phosphate* | Lactulose* |
| Levoglucosan** | Maltotriose** | Glucoheptose* | Lactamide* |
| Lactulose* | Isoleucine* | Galactose-6-phosphate** | Inositol-4-monophosphate** |
| Lactose* | Glyceric acid* | Dopamine** | Inosine-5-monophosphate* |
| Isoheptadecanoic acid** | 3-phenyllactic acid* | Docosahexaenoic acid** | Glycine* |
| Glycerol-alpha-phosphate** | 2-aminobutyric acid* | Dihydrocholesterol* | Glycerol-3-galactoside** |
| Glycerol-3-galactoside** | 1-kestose** | Dehydroascrobic acid** | Glucose-6-phosphate* |
| Glycerol* |  | Citric acid* | Glucose** |
| Glyceric acid** | **Age × Cattle** | Cholesterone** | Fumaric acid* |
| Glutamate** | Xylitol* | Cholesterol* | Fructose* |
| glucose-1-phosphate** | Xanthosine* | Cellobiose* | Docosahexaenoic acid* |
| Fructose** | Valine** | Campesterol* | Cholesterol* |
| Erythritol** | Uridine* | Butyrolactam* | Cellobiose* |
| Dihydroxyacetone** | Uric acid** | Beta-glycerolphosphate* | Beta-glycerolphosphate* |
| Cysteine** | Tyrosine** | Arachidonic acid** | Asparagine** |
| Butane-2,3-diol** | Tryptophan** | Adenosine-5-monophosphate** | Adenosine-5-monophosphate** |
| Aspartic acid** | Threonine** |  |  |
| Asparagine** | Threonic acid* | 4-hydroxybutyric acid* | 5-aminovaleric acid** |
| Alanine** | Succinic acid* | 2,5-dihydroxypyrazine** | 4-hydroxybutyric acid** |
| 9-myristoleate* | Shikimic acid** | 1-monostearin* | 2-picolinic acid* |
| 5-aminovaleric acid* | Serine** |  |  |
| 4-hydroxyphenylacetic acid** | Ribose-5-phosphate* | **Age × Liver** | **Age × Cattle × Liver** |
|  | Ribono lactone* | Tyramine* | Glutamine** |
| 3-phosphoglycerate** | Proline** | Succinic acid* | Fructose-6-phosphate* |
| 3-phenyllactic acid** | Phosphoethanolamine** | Sophorose* | Cystein sulfonic acid* |
| 3,6-anhydro-D-galactose** | Phenylalanine** | Shikimic acid* | Creatinine* |
| 2-picolinic acid* | Oxoproline* | Putrescine* | 3-hydroxybutyric acid* |
| 2-monoolein** | N-methyl-UMP** | Oxoproline* |  |
| 2-hydroxyisocaproic acid** | Nicotinamide* | Ornithine* |  |
| ^1^Main effect and interaction classifications: Age = 7 d or 35 d age duration; Cattle type = Dairy-cross or Native; Liver = Major liver abscess (A+, Elanco Animal Health Liver Check System) or Edible liver (healthy)  **P* < 0.05  ***P* < 0.01 | | | |
